# Adult Active Transport in the Netherlands: An Analysis of Its Contribution to Physical Activity Requirements

**DOI:** 10.1371/journal.pone.0121871

**Published:** 2015-04-07

**Authors:** Elliot Fishman, Lars Böcker, Marco Helbich

**Affiliations:** 1 Department of Human Geography and Spatial Planning, Faculty of Geosciences, Utrecht University, Utrecht, The Netherlands; 2 Department of Innovation, Environmental & Energy Sciences, Faculty of Geosciences, Utrecht University, Utrecht, The Netherlands; CNRS, FRANCE

## Abstract

**Introduction:**

Modern, urban lifestyles have engineered physical activity out of everyday life and this presents a major threat to human health. The Netherlands is a world leader in active travel, particularly cycling, but little research has sought to quantify the cumulative amount of physical activity through everyday walking and cycling.

**Methods:**

Using data collected as part of the Dutch National Travel Survey (2010 – 2012), this paper determines the degree to which Dutch walking and cycling contributes to meeting minimum level of physical activity of 150 minutes of moderate intensity aerobic activity throughout the week. The sample includes 74,465 individuals who recorded at least some travel on the day surveyed. As physical activity benefits are cumulative, all walking and cycling trips are analysed, including those to and from public transport. These trips are then converted into an established measure of physical activity intensity, known as metabolic equivalents of tasks. Multivariate Tobit regression models were performed on a range of socio-demographic, transport resources, urban form and meteorological characteristics.

**Results:**

The results reveal that Dutch men and women participate in 24 and 28 minutes of daily physical activity through walking and cycling, which is 41% and 55% more than the minimum recommended level. It should be noted however that some 57% of the entire sample failed to record any walking or cycling, and an investigation of this particular group serves as an important topic of future research. Active transport was positively related with age, income, bicycle ownership, urban density and air temperature. Car ownership had a strong negative relationship with physically active travel.

**Conclusion:**

The results of this analysis demonstrate the significance of active transport to counter the emerging issue of sedentary lifestyle disease. The Dutch experience provides other countries with a highly relevant case study in the creation of environments and cultures that support healthy, active living.

## Introduction

Modern, urban lifestyles have engineered physical activity (PA) out of everyday life and this has resulted in an emerging, widespread threat to population health caused by sedentary lifestyles [1–3]. It is estimated that physical inactivity causes 21–25% of the global burden of disease from breast and colon cancer and even greater proportions for diabetes (27%) and ischaemic heart disease (30%) [4]. Physical activity is increasingly regarded as the *‘best buy’* in preventative health measures [5] and walking and cycling represent one of most effective methods of building PA into daily life [6], whilst at the same time reducing CO_2_ emissions, air pollution and congestion caused by motorised forms of transport [7].

The Netherlands is widely regarded as a world leader in active transport, particularly cycling [8] with 16% of the total road network dedicated to cycle paths [9]. A third of all trips under 7.5 km are cycled [10]. The Dutch reputation for cycling, combined with relatively strong walking levels therefore presents what might be considered world’s best practice levels of PA through incidental, transport based walking and cycling. Yet despite the fact 27% of all trips are by bicycle [11], it is reported some 44% of the Dutch population over 12 years old do not engage in sufficient levels of PA to protect against sedentary lifestyle diseases [12].

The objective of this paper is to quantify walking and cycling’s contribution to meeting minimum adult PA guidelines in the Netherlands. By investigating the Dutch National Travel Survey (NTS) data for the period 2010 to 2012, fundamental socio-demographic, spatial and environmental factors associated with higher and lower levels of active transport are determined. In doing so, the World Health Organisation’s Global Recommendations on Physical Activity for Health [13] are used to provide the foundation for the PA levels recommended for healthy living. For healthy adults aged between 18–64 years it is recommended to engage in a minimum of 150 minutes of moderate intensity aerobic activity throughout the week [13] *or* an equivalent combination of moderate and vigorous intensity activity. Bicycle riding has been found to achieve the necessary intensity to qualify for moderate-intensity activity [14–17]. To operationalize the PA requirements, an alternative and underexplored approach translating transport data into metabolic equivalents of tasks (METs), i.e. a measure representing the energy cost of physical activities, has been undertaken, and explained in more detail in Section 3.1.

This study provides a unique contribution to the literature and fills an important research gap in three crucial respects. Firstly, the analysis includes walking and cycling to and from public transport, which is significant in the Netherlands (e.g. 40% of trips to train stations are by bike) [10] but rarely included in travel diary analyses [18,19]. Secondly, the level of active transport found in this analysis is assessed against minimum PA requirements, to determine the degree to which Dutch walking and cycling contributes to meeting PA recommendations for healthy living. Thirdly, the relationships between active transport and personal and household socio-demographics, transport resources, the built environment, as well as weather conditions are identified. This analysis provides researchers and policy makers with a detailed quantification regarding the Dutch experience of integrating PA into everyday life through active transport, to assist policy makers and practitioners address the growing issue of sedentary lifestyle disease.

The rest of this paper is structured as follows. Section 2 provides a brief review of the literature, Section 3 outlines the methodological approach and Section 4 provides a description and discussion of the results. Section 5 concludes the paper by highlights how major findings relate to the existing literature, some implications of the study and identifies areas for future research.

## Literature Review

Analyses of national travel survey and census data have taken place in a number of countries, including some that have focused primarily on changes to active mode usage over time. Pucher et al. [20] conducted an examination of changes in walking and cycling between 2001 and 2009, based on data from the United States National Travel Surveys. They found a slight increase in walking levels (an extra nine miles on average, per year) and an even more modest increase in cycling (an extra five miles, per year). Walking at least 30 minutes per day increased from 7.2% to 8.0% in 2009. Only 1.7% recorded any cycling and 0.9% recorded at least 30 minutes of cycling per day. These figures remained unchanged between 2001 and 2009.

For England and Wales it is reported that approximately 11% and 3% of the population walked or cycled to work in 2011 respectively, and these levels were essentially unchanged from ten years earlier [21]. Interestingly, and unlike other forms of PA, those of a lower socio-economic status were found to have slightly higher levels of active transport—although the author notes this trend is decreasing, especially for cycling.

In Australia, journey to work data is collected via the Census [22]. Previous analysis has shown that approximately 4% of trips to work are on foot [23] and Rissel [24] shows that rates of cycling to work were stable at around 1% between 1986–2006. A recent analysis by Loader [25] showed a small increase in cycling to work in Australian capital cities between 2006 and 2011, although geographic variation was considerable [25]. However, the Australian Census is collected on a single day every five years, in winter. Rain or other climatic conditions adverse to walking and cycling can therefore distort the results (e.g. see [19,26,27,28]).

Several studies investigate active transport patterns in the Netherlands using data collected by the Dutch National Travel Survey (NTS) [29]. Harms et al. [30] report that although overall Dutch cycling shares have been relatively stable at around 27% over decades, cycling distances have increased by 14%. Just over half of all trips for those up to 18 years of age are by bicycle and this rises to 60% when focused solely on trips to school [30]. However, almost all of the 14% growth in the distance cycled is due to those aged 50 years or more. Another trend picked up by Harms et al. is a reduction in car use by those aged 18–30, consistent with evidence emerging from other western countries [31,32]. Women have a higher share of cycling as a proportion of all trips but men cycle greater distances. Income has no effect on the proportion of trips cycled but those with higher income reported longer trips by bike. Moreover, cycling has increased in cities and decreased in rural areas.

In another Dutch study Scheepers et al. [6] examined exclusively short trips (under 7.5 km) using NTS data collected between 2004–2009. Walking and cycling mode share is analysed together in comparison to car use for different trip purposes (i.e. shopping, commuting, chauffeuring, and sports) and Scheepers et al. found that for trips under 7.5 km, 44% are made by car, regardless of trip purpose. Bicycle use was highest for commuting (47%), falling to 35% for both chauffeuring and shopping. Walking was highest for chauffeuring, accounting for 21% of trips, reducing to 9% for commuting. Trip durations were found to be similar, on average, between car, bicycle and foot (around 10 minutes) although distance travelled varies from 3.3 km for car, 2.1 km for cycling and 0.8 km for walking.

In summary, this brief literature review illustrates that the Netherlands has comparatively very high rates of active travel. Differences in participation levels can be seen based on factors such as income, working hours, age and trip purpose. The present study compliments these existing active transport studies (e.g. see [6,30]) in a number of ways. Firstly, the present paper provides a detailed analysis of the level of PA necessary for healthy living, currently underexplored in transport studies. Secondly, in addition to providing a general account of active transport, we include in our empirical analyses walking *and* cycling separately, which is important given the significant health benefits of both [18,33]. Moreover, trip segments that involve walking or cycling to/from public transport have also been considered. The present study provides a cumulative analysis of active travel, in order to determine the quantity of PA provided by active transport patterns. The importance of including these *trip stages* is underlined by the fact that some 40% of train journeys in the Netherlands start with a bicycle trip [10]. Thirdly, this research complements existing studies by including trips over any distance, which is important because some 15% of trips between 7.5 km–15 km are by bicycle in the Netherlands [10]. To restrict the analysis to trips under 7.5 km, as in the case of Scheepers et al. [6] would therefore leave a great deal of PA unreported. Finally, this study combines data over the three most recently available years (2010–2012).

## Materials and Methods

The following section introduces the datasets and describes the methodological approach taken to quantify the contribution to PA guidelines from Dutch active travel.

### Data

The Dutch NTS (2010–2012) is the prime data source and is provided by Onderzoek Verplaatsingen in Nederland [34], which contains travel diary data on transport modes, trip frequencies and durations. Participants are asked to record every trip, including trip stages, during the course of one day and this day is randomised to cover the full calendar year (to take weather effects into account, for instance). The participants record their trips using a travel diary that has been mailed in hardcopy. The sample selected for this study only includes participants that recorded a trip during the day of the travel diary survey and only those 18 years or older. Furthermore, after pre-screening the data, cases with missing data are excluded from subsequent analyses. The sample represents 74,465 individuals and 239,929 trips distributed across all regions of the Netherlands and is reflective of the Dutch general population. For public transport journeys, any active transport trip segment (e.g. getting to/from a train station by foot or bicycle) has also been included in the analysis.

Instead of operationalizing mode choice as the dependent variable directly, we have focused on metabolic equivalents of tasks (METs) for walking and cycling. Metabolic equivalent of tasks are a standard method used to assess the energy cost of different physical activities and is defined by Ainsworth et al. [15] as the ratio of the activity metabolic rate to the metabolic rate at rest. Rest is considered to have a MET value of one [35]. One MET is defined as 1 kcal/kg/hour. Ainsworth et al. [15] provide a compendium of MET values for different types of walking and cycling. The MET values for walking and cycling in this paper have used the Ainsworth et al. (2011) values, adjusted to the Dutch walking and cycling speeds of 5 km/h and 15 km/h respectively acquired from the data, resulting in MET values of 5.8 for cycling and 3.8 for walking. MET hours for walking and cycling were derived by multiplying daily hours engaged in walking and cycling by the corresponding MET values.

In addition to trip-specific data represented as METs, the NTS provides several variables describing personal (e.g. age, gender, ethnicity, education) and household characteristics (e.g. income), as well as transport resources (e.g. number of cars per household, bicycle ownership). Furthermore, each respondent’s mobility record has been linked to a meteorological record for that specific day from one of the 36 Royal Dutch Meteorological Institute weather stations closest to the residential postal code. We include daily measures of the maximum air temperature (in °C), precipitation sum (in mm) and average wind speed (in m/s)—the three most commonly used meteorological variables in existing studies (e.g see [19,26]). Finally, using a Geographic Information System, the respondents’ residential locations are geocoded on a 4-digit postal code level. This allows computation of the following proxy variables describing the built environment: (1) residential address density (in thousand addresses per km^2^) aggregated per 4-digit residential post code has been derived from the ‘Basisregistratie Adressen en Gebouwen 2012’; (2) land use diversity operationalized based on the Shannon diversity index of main land use classes; and the surface-area percentages of (3) green space and (4) water, abstracted from the 2007/08-dataset ‘Landelijk Grondgebruiksbestand Nederland’. Table 1 provides an overview and some descriptives about the data and S2 Table provides background data on address density and land use diversity.

**Table 1:** Data description and descriptive statistics.

		Min.	Max.	Mean	Std. dev.	%
*METs values*						
Walking as main mode		0.0	129.5	0.687	2.092	
Cycling as main mode		0.0	282.8	1.344	4.161	
Walking/cycling stages to public transport		0.0	41.6	0.102	0.591	
Total		0.0	282.8	2.133	4.628	
*Individual and household characteristics*						
Age (in years)		18.0	99.0	48.447	16.539	
Gender	Male					48.8
Ethnicity	Non-western					5.3
Occupation	Work >30h					43.9
	Work 12–30h					17.7
	Domestic					7.3
	Student					5.5
	Unemployed					2.0
	Unfit to work					2.6
	Retired					21.0
Education	Low					30.4
	Middle					38.0
	High					31.6
Net annual household income	<€20K					12.6
	≥€20<40K					43.0
	≥€40K					44.4
*Transport resources*						
Cars per household		0.0	10.0	1.362	0.836	
Driver’s license owners (%)						88.0
Bicycle ownership						91.7
Moped ownership						5.4
*Built environment*						
Address density (in 1000 addresses / km^2^)		0.0	11.4	1.411	1.409	
Land use diversity		0.0	2.8	1.776	0.492	
Surface area percentage green		2.7	97.8	55.162	22.221	
Surface area percentage water		0.0	76.4	5.624	7.133	
*Weather variables*						
Daily max. air temperature		-9.0	35.9	13.396	7.554	
Daily precipitation sum		0.0	142.3	2.201	4.652	
Daily average wind speed		0.4	16.3	4.112	1.921	

### Statistical analysis

The Tobit regression model [36,37] was used for the multivariate analyses. The dependent variables, expressed in MET-hours gained from various forms of active transport per person per day, are all ratio variables that (1) cannot be negative, and that (2) shows an excess of zeros—as a consequence of a relatively large share of respondent’s not performing trips by active modes on the day of the survey. Under these conditions a standard ordinary least squares regression would not be appropriate and wrongly assumes that data are not censored to a certain value and would wrongly predict non-existing negative value. For these reasons a Tobit model is preferred.

The model introduces an unobservable latent dependent variable that is predicted by a set of independent variables via their respective coefficients. It runs under the condition that the observed variable is equalled to the latent variable whenever this latent variable is positive, or equalled to zero whenever the latent variable is negative. Some respondents may have their residential locations within the same postal codes, thereby sharing the same spatial background and thus violating the independence assumption between the observations. To account for intragroup correlations—i.e., interdependencies between respondents located within one postal code—the standard errors and variance–covariance matrices are adjusted using the 4-digit residential postal code as grouping variable. To estimate and compare the quality of the Tobit models, the McKelvey-Zavoina pseudo R^2^ will be used, as recommended by Veall and Zimmermann [38]. This gives a better approximation of the model’s quality than regular pseudo R^2^ measures, such as McFadden’s.

## Results and Discussion

### Descriptive statistics

A little over half (57%) the sample did not record any walking or cycling on the day of the survey, yet due to those that did, on average, the sample still participated in more than the recommended minimum level of PA. This suggests that for those who do *any* walking or cycling, they are likely to exceed the minimum daily guidelines for PA by a considerable margin.

The quantity of walking and cycling, both as single mode travel, as well as in conjunction with public transport has been calculated and shows men and women gain 41% and 55% *over* the recommended minimum level of PA respectively from walking and cycling alone. This is based on internationally recognised minimum level of PA: 10 MET-hours per week [12], corresponding to 1.42 MET-hours per day. Some 38% of Dutch adults recorded sufficient levels of active travel to meet or exceed recommended minimum levels of PA.

For single mode trips, Dutch adults cycle about 3.5 km (men) and 3.7 km (women) per day and between 0.9 km (men) and 1.0 km (women) on foot. An average of 185 m of combined walking and cycling is estimated to be travelled to connect to/from public transport. Dutch men engage in an average of 24 minutes of active travel per day, compared to about 28 minutes for women. See S1 Table for additional information.

**Fig 1:**
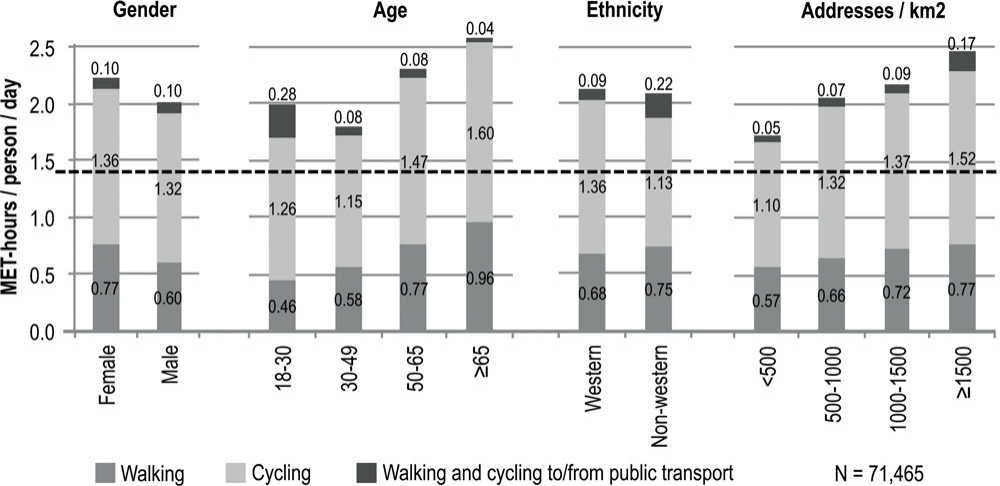
MET-hours gained from daily active transport for different population categories and spatial contexts. NB: Dashed line represents the minimum recommended level of PA.

Fig 1 provides an illustration of MET-hours per person per day from active transport, using four variables; gender, age, ethnicity and address density. As previously described, women engage in greater levels of active transport than men. Those over 65 years of age achieve the highest level of active transport, followed by those aged between 50–65 years. To some degree this may reflect a bias in our data, whereby only those who left the house on the day of the travel survey were included in the analysis. It is however interesting to note that people aged 18–30 participate in considerably more active travel in conjunction with public transport than other age groups. Those of a non-western ethnicity engage in more walking and less cycling (single mode), but more active travel to/from public transport than those of a western ethnicity. Overall, active travel levels are similar between both groups. Address density has a positive effect on METs, i.e. the more addresses in a given area, the greater the levels of active transport are likely to be, for each of walking and cycling (single modes) as well as when travelling to/from public transport.

### Multivariate regression analysis

This section reports the estimation results of four Tobit models where MET-hours for different active transport modes are regresses upon the explanatory variables shown in Table 1. Overall, the estimated Tobit models show a good fit. This is confirmed by the significant likelihood ratio Chi^2^ values and the McKelvey-Zavoina pseudo *R* ^*2*^ measures, which range from 0.054 for the walking model to 0.225 for the cycling model. The remaining section discusses the factors significantly associated with active transport. Variables with a *p*-value below 0.05 are considered to be statistically significant. Table 2 provides the estimated coefficients and associated significance values for each model.

**Table 2:** Estimation results of the Tobit model for different modes of active transport (MET-hours per person per day gained from active transport (AT)).

		MET-h walking		MET-h cycling		MET-h AT to/from PT		MET-h AT total	
		Coef.	t-value	Coef.	t-value	Coef.	t-value	Coef.	t-value
Intercept		-2.694	-9.04***	-16.540	-19.25***	-.751	-2.17*	-1.883	-5.99***
*Socio-demographics*									
Age (in years)		.011	3.90***	.014	3.08**	-.026	-6.89***	.012	3.74***
Male (D)		-.638	-10.41***	.136	1.31	-.306	-4.02***	-.221	-3.22**
Non-western ethnicity (D)		-.072	-.61	-2.002	-7.06***	.805	6.78***	-.379	-2.60**
Occupation (ref. work >30h)	Work 12–30h (D)	.789	10.23***	2.149	13.02***	-.569	-5.15***	1.489	13.96***
	Domestic (D)	1.666	15.32***	2.488	11.87***	-1.913	-9.85***	2.174	15.10***
	Student (D)	-.023	-.17	3.248	11.56***	2.906	17.69***	2.960	15.16***
	Unemployed (D)	1.531	8.96***	2.117	6.92***	-.758	-3.13**	2.097	10.53***
	Unfit to work (D)	1.568	10.29***	.813	2.61**	-1.164	-4.83***	1.336	6.54***
	Retired (D)	1.375	13.33***	1.259	6.79***	-1.059	-6.39***	1.601	12.22***
Education (ref. higher)	Low (D)	-.506	-6.82***	-.577	-4.64***	-1.269	-11.78***	-.752	-8.85***
	Middle (D)	-.223	-3.43**	-.751	-6.82***	-.790	-9.39***	-.684	-9.10***
Net annual hh-income	<€20K (D)	.073	.82	-.985	-5.69***	-.991	-7.61***	-.623	-5.61***
	≥€20<40K (D)	.153	2.45*	-.538	-4.92***	-.804	-9.09***	-.273	-3.76***
*Transport resources*									
Cars per household		-.475	-10.87***	-2.229	-18.56***	-1.156	-13.96***	-1.594	-21.97***
Driving license (D)		-.563	-6.73***	-1.740	-9.73***	-1.803	-15.82***	-1.839	-14.54***
Moped ownership (D)		-.036	-.27	-2.039	-9.36***	-.687	-4.17***	-1.219	-8.48***
Bicycle ownership (D)		-.037	-.41	14.356	22.48***	-.095	-.83	4.185	27.50***
*Built environment*									
Addresses*1000 / km^2^		.211	7.01***	.319	6.59***	.181	5.25***	.353	10.76***
Land use diversity		.209	2.91**	-.015	-.12	-.245	-2.27*	.116	1.41
Surface area % green		-.013	-6.47***	-.008	-2.37*	-.008	-2.90**	-.012	-5.66***
Surface area % water		-.006	-1.60	-.023	-3.10**	.021	4.59***	-.009	-2.01*
*Weather variables*									
Daily max. air temperature		-.032	-9.090***	.143	19.52***	-.011	-2.51*	.054	13.61***
Daily precipitation sum		-.016	-2.660**	-.060	-6.08***	-.014	-1.87	-.046	-6.99***
Daily average wind speed		-.024	-1.740	-.128	-5.00***	.027	1.43	-.087	-5.39***
Model fit:									
Likelihood ratio Chi^2^(df.)		2393.591(24)	***	7234.417(24)	***	4915.755(24)	***	6749.059(24)	***
Ps. R^2^ (McKelvey-Zavoina)		.054		.225		.201		.113	

Significance levels:

‘***’ < 0.001;

‘**’ < 0.01;

‘*’ < 0.05

(D) Dummy Variable.

### Socio-demographic factors

The results of the model show that as age increases, the likelihood of walking and cycling increases, confirming the relationship found in Section 4.1. This differs to some extent from the results of Harms et al. [30], as their study included those under 18 years of age, who were found to have substantially higher levels of cycling than all other age groups. The present study found the age/active travel relationship is reversed however when focused on walking and cycling to/from public transport (i.e. younger age groups are more likely to use active transport to connect with public transport). Dutch adult women are more likely to walk than men, but no gender relationship was detected for cycling. Additionally, Dutch adult women were more likely than men to accumulate PA by active travel to/from public transport. This is in sharp contrast (certainly with regard to cycling) from studies in the US [39] and Australia [40], but does confirm the descriptive statistics in Section 4.1.

Cycling is negatively associated for those of non-western ethnicity, confirming previous studies (e.g. see [9,30,41]), but no relationship with ethnicity was detected for walking. Interestingly, and not identified in previous studies, those of a non-Western background were more likely to participate in active travel as part of public transport journeys.

People working < 30 hours per week had a greater likelihood of walking and cycling as single mode trips but less likely to engage in active transport to/from public transport. Students were much more likely to cycle than non-students. Interestingly, the unemployed (including those unfit for work), as well as retirees walked and cycled more than employed persons, although this may be explained by only including those that recorded some travel within our analysis. The relationship with employment was reversed for active travel as part of a public transport trip. Both higher income and higher education have a positive effect on active transport. The education effect is in congruence with earlier studies finding higher active mode share for highly educated people (e.g. see [42]). The income effect supports the positive relationship between income and cycling found in Sydney [43] and Brisbane [44], but contrasts somewhat with other Dutch research by Dieleman et al. [42] who found a negative effect of income on walking and cycling mode share and Harms et al. [30], who found the proportion of trips by bicycle doesn’t appear to differ by income. It should be noted that in this study, we measured minutes spent cycling, rather than the proportion of all trips and this may well explain the difference between the results in this study and that of Harms et al. [30].

### Transport resources

When looking at the relationship between walking and cycling and transport resources (e.g. car ownership), the results are generally intuitive. More cars in the household and holding a drivers license have negative effects not only on cycling, as is often discussed in the literature (e.g. see [30,45]), but also on walking and active transport as part of trips made by public transport. Given the strength of this relationship between car ownership and active transport, the global phenomenon of young people not aspiring to gain their drivers licence in the manner of previous generations [31] points to the potential for greater levels of walking and cycling in the future. In addition to car ownership, moped ownership was negatively associated with cycling, although no significant effect of moped ownership was found on walking levels. Given the relatively short distances of many moped journeys (around 6 km on average according to the NTS 2010–2012) there may be some public health benefit in seeking to shift some moped trips to cycling, particularly given that mopeds negatively impact on air quality [46]. One of the strongest and most logical relationships in the analysis was that between bicycle ownership and use. However, the relationship between owning a bicycle and active travel to/from public transport was not significant. Whilst this might seem counter-intuitive, it is simply a product of those without a bike walking more.

### Urban form

In relation to urban form characteristics, residential address density was positively related to time spent walking and cycling. This is generally consistent with the literature and the descriptive data in Section 4.1. In a recent review, Giles-Corti et al. [47] found that those residing in higher density areas walked and cycled more than those in lower density suburbs. Overweight and obesity was also found to increase as density reduced [47]. Ewing et al. [48] found that density was negatively associated with common diseases of a sedentary lifestyle, such as diabetes and hypertension, but were unable to detect a relationship with PA, which, as the authors note, may be due to active travel not being included in the PA calculation. Research in Atlanta, United States found that land use mix was strongly associated with reductions in obesity, at the individual level [49].

Greenness has a negative effect on active mode usage. This may be surprising at first glance, because one may expect it to be more pleasant to cycle in green areas. A lack of greenness may however simply be acting as an additional proxy for highly urbanised areas, which in the Netherlands due their compact designs, short distances and car-unfriendliness are relatively attractive for walking and cycling.

### Weather

Another important spatiotemporal context for active transport is formed by weather conditions. In congruence to existing international (e.g. see [50,51,52]) as well as Dutch studies (e.g. see [19,53,54,55]) precipitation sum and wind speed were found to have negative effects on active transport. Cycling appears to be affected more strongly than walking. A likely explanation could be that cyclists are more exposed to wind than pedestrians and have fewer opportunities to take shelter against rain inside or along buildings or by usage of umbrellas. In line with the above-mentioned studies, maximum daily air temperature has a positive effect on cycling. In contrast, a negative temperature effect is observed on walking, which contradicts earlier findings of a non-significant effect in Rotterdam, the Netherlands [54] or a positive effect in Montreal [56]. Nevertheless, higher air temperatures have a net positive effect on MET-hours gained from combined cycling and walking overall.

## Conclusion

Physical inactivity is a significant and growing contributor to the burden of disease [13]. Active transport is increasingly seen as an important opportunity to counteract the incidence of sedentary lifestyle diseases (e.g. diabetes, obesity) [6], and one that can be integrated into everyday life [21,35]. This paper has assessed levels of active transport among Dutch adults via an analysis of the NTS data from 2010–2012, and crucially, converts this activity into MET-hours. The main objective of the paper was to determine the degree to which active transport in the Netherlands contributes to PA requirements.

The empirical findings have revealed that Dutch men and women, on average, gain 41% and 55% more PA respectively than minimum recommended levels. Overall, 38% of Dutch adults meet or exceed recommended minimum levels of PA from active travel alone. However, approximately half the investigated sample recorded no active transport at all. This means that those who did any walking or cycling are likely to be achieving PA levels significantly higher than recommended minimums. Women were found to engage in slightly higher levels of active travel than men, and this contrasts with other developed countries such as the United States [20] and Australia [23]. The Tobit regression model highlighted that several significant factors are associated with greater levels of active transport, including older age, native Dutch ethnicity (for cycling, reversed for walking), and not working more than 30 hours per week. Owning a car was negatively associated with active travel and moped ownership reduced the likelihood of cycling. Higher address densities were positively associated with active travel and rain and wind speed were negatively associated with active transport in general and cycling in particular.

This paper has several key strengths. Firstly, combing the three most recently available years of the Dutch NTS provides a large and representative sample of the Dutch population, and encompasses all trips, staggered throughout the year to control for such issues as meteorological variation. Secondly, trips to and from public transport have been included and this is a distinct advance on previous studies focused on walking and cycling, in which these *trip stages* are often omitted. Thirdly, and perhaps most crucially in terms of an original contribution to the literature, this paper has converted PA gained via active travel into METs and compared this to established minimum requirements. Finally, the application of the Tobit model has offered an analytical approach to identifying factors associated with higher levels of active travel, while accounting for the large number of zeros in the data associated with non-active travel.

There are however some limitations to this study that could be addressed in further research. Firstly, the data is self-reported which might impact on its accuracy. Previous analysis has found differences between self-reported and accelerometer-measured data, particularly with overweight individuals [57]. Inspection of the dataset reveals a tendency for respondents to the travel diary survey to approximate their choice to the nearest ‘round’ number (e.g. 5 min, 10 min). A further limitation is that people’s actual physical intensity (measured in METs) will vary depending on body mass, age, type of bicycle, and items they may carry. This analysis has taken well-established values for METs based on average travel speeds. Whilst this is the best method available to convert travel data into energy costs, it does not account for individual variation. To account for these issues, further research may triangulate travel diary data with data from physiological measurement devises attached to each subject. Additionally, our analysis has only included those who reported making at least one trip during the day of the survey. However, some 15%- 20% recorded no travel on the day of the survey and these people have not been included in the analysis, nor have people less than 18 years old. An important area of future research concerns those who recorded no active travel, as these individuals may be at greater risk of developing sedentary lifestyle disease.

This paper has drawn an assumption that all reported trips result from travelling from one place to another. There may however be instances when an activity of a purely recreational nature was included in the diary. This does not impact on the validity of the results, as PA benefits still accrue, but it is nevertheless important to clarify. Furthermore, the NTS does not discriminate between traditional bicycles and e-bikes. E-bikes are becoming more popular in the Netherlands [58] and although their use provides PA, it is less intensive than the same journey undertaken on a traditional bike [14,17,59]. Future research is required investigating the impacts of e-bikes on travel behaviour. The NTS is a diary-based snapshot of transport activity for one day only, and this may not be representative of typical travel throughout the week. Finally, there are limits to the generalizability of the results to other countries given that Dutch rates of active transport are significantly higher than almost all other jurisdictions [8].

There are several policy implications arising from this research. Firstly, Dutch active transport is having a significant influence on population level PA levels. Therefore, other countries may benefit from examining Dutch transport policies, in order to determine whether there are opportunities to adopt or adapt some of these within their transport policy environment. Whilst this study did not investigate the reasons why the Dutch have significantly higher rates of active transport than other developed countries, there are several plausible explanations that have implications for other jurisdictions. Past research suggests the bicycle infrastructure policies of separating bicycles and motor vehicles when speeds exceed 30 km/h may be an effective approach to reducing current barriers to cycling [8,41]. Moreover the Dutch emphasis on compact, mixed use development and an integrated system of public transport and cycling have all helped achieve strong levels of active transport in the Netherlands [60]. Parking, speed and movement restrictions placed on motor vehicles in many Dutch cities have also worked to minimise the competitive advantage of car use over active travel. These Dutch transport and land use policies serve to support the use of active transport. With the rapid rise of sedentary lifestyles and the consequent increase in non-communicable diseases, the Dutch experience provides other countries with a highly relevant case study in the creation of environments and cultures that support healthy, active living.

## Supporting Information

S1 Table Cycling and walking duration, speed, distance and METs. (DOCX)

S2 Table Spatial data. (TXT)
