# Supplementary material for: Adult Active Transport in the Netherlands: An Analysis of Its Contribution to Physical Activity Requirements
Source: PLoS One. 2015 Apr 7;10(4):e0121871. doi: 10.1371/journal.pone.0121871 (PMC4388541; doi:10.1371/journal.pone.0121871)
Supplement: S1 Table — (DOCX) [file pone.0121871.s001.docx]

# Supporting Information Legends

Table S1: Cycling and walking duration, speed, distance and METs

|  | **Men** | **Women** |
| --- | --- | --- |
| Bicycling duration (min) per person, per day (mean) | 14.0 | 14.7 |
| Walking duration (min) per person, per day (mean) | 10.3 | 13.1 |
| Average speed bicycling (km/h) | 15.0 | 15.0 |
| Average speed walking (km/h) | 5.0 | 5.0 |
| Bicycling distance (km) per person, per day (mean) | 3.5 | 3.7 |
| Walking distance (km) per person, per day (mean) | 0.9 | 1.1 |
| MET value for cycling | 5.8 | 5.8 |
| MET value for walking | 3.8 | 3.8 |
| MET Hours per person, per day (cycling, single mode) | 1.3 | 1.4 |
| MET Hours per person, per day (walking, single mode) | 0.6 | 0.8 |
| MET Hours per day total active transport* | 2.0 | 2.2 |

*Includes both single mode active transport trips and those made in conjunction with public transport.
